# Symmetry collapse due to the presence of multiple local aromaticity in Ge_24_^4−^

**DOI:** 10.1038/s41467-022-29626-5

**Published:** 2022-04-20

**Authors:** Hong-Lei Xu, Nikolay V. Tkachenko, Dariusz W. Szczepanik, Ivan A. Popov, Alvaro Muñoz-Castro, Alexander I. Boldyrev, Zhong-Ming Sun

**Affiliations:** 1 State Key Laboratory of Elemento-Organic Chemistry, Tianjin Key Lab of Rare Earth Materials and Applications, School of Materials Science and Engineering, Nankai University, Tianjin, China; 2 Department of Chemistry and Biochemistry, Utah State University, Logan, UT USA; 3 Department of Theoretical Chemistry, Faculty of Chemistry, Jagiellonian University, Kraków, Poland; 4 Department of Chemistry, The University of Akron, Akron, OH USA; 5 Grupo de Química Inorgánica y Materiales Moleculares, Facultad de Ingeniería, Universidad Autonoma de Chile, El Llano Subercaseaux, Santiago, Chile

**Keywords:** Inorganic chemistry, Physical chemistry, Coordination chemistry

## Abstract

Understanding the structural changes taking place during the assembly of single atoms leading to the formation of atomic clusters and bulk materials remains challenging. The isolation and theoretical characterization of medium-sized clusters can shed light on the processes that occur during the transition to a solid-state structure. In this work, we synthesize and isolate a continuous 24-atom cluster Ge_24_^4−^, which is characterized by X-ray diffraction analysis and Energy-dispersive X-ray spectroscopy, showing an elongated structural characteristic. Theoretical analysis reveals that electron delocalization plays a vital role in the formation and stabilization of the prolate cluster. In contrast with carbon atoms, 4 s orbitals of Ge-atoms do not easily hybridize with 4p orbitals and s-type lone-pairs can be localized with high occupancy. Thus, there are not enough electrons to form a stable symmetrical fullerene-like structure such as C_24_ fullerene. Three aromatic units with two [Ge_9_] and one [Ge_6_] species, connected by classical 2c-2e Ge-Ge σ-bonds, are aligned together forming three independent shielding cones and eventually causing a collapse of the global symmetry of the Ge_24_^4−^ cluster.

## Abstract (web summary)

Gaining insight on the structural transformations from atomic clusters to bulk materials is challenging. Here the authors synthesize a continuous cluster of germanium Ge_24_^4−^, which can be viewed as two terminal Ge_9_ units bridged via a Ge_6_ central fragment, and characterize it by several techniques including X-ray diffraction; theoretical analysis indicates the presence of three aligned independent aromatic fragments.

## Introduction

Understanding how the addition of atoms one by one leads to the transition from a single atom to a diatomic molecule to atomic clusters and finally to the formations of bulk solid-state allotropes is a dream of many chemists. This understanding will help us to design tailorable materials with ever unusual structures and other physical and chemical properties. Today we still do not understand how such evolution is happening. A striking example is carbon—one of the most investigated elements. Although it is known that the transition from diatomic C_2_ to larger carbon clusters goes through the formation of linear chains^1^, cyclic structures^2^, and cage-like fullerenes^3^, we still do not completely know how fullerenes will transform upon further addition of atoms and finally form bulk graphite or diamond. For other elements, our knowledge of this evolution is less clear. Even for the most similar isoelectronic elements of the IV group of the Periodic Table (Ge and Si), computational studies showed that atomic clusters’ structures behave differently upon growth^4–6^. Thus, the smallest fullerene-like structure for carbon atom occurs at 20 atoms^7^ and continues to evolve beyond. However, according to the computational results, Si and Ge tend to form prolate structures for medium-sized clusters rather than spherical-like fullerenes. The experimental evidence of such behavior so far was limited to ion mobility experiments^8^ and 2D electron microscopy experiments^9^. Although theory can propose some trustworthy candidates for low energy structures, one of the most reliable pieces of experimental evidence—a solid state X-Ray characterization, is still lacking for large continuous Ge clusters. Hence the isolation of medium-sized pure germanium species as a key intermediate to understand the structural transition is of greatest importance. The isolated ligand-free germanium clusters with over 10 atoms known to date always exhibited a sole coupling model of small clusters^10–12^, which should be better regarded as polymerization tendency. In addition, although the silyl-protected Ge_18_[Si(SiMe_3_)_3_]_6_ cluster cannot be seen as polymers like [Ge_9_-Ge_9_]^6−^, the outer ligands may dramatically affect the structures of cluster cores and thus it cannot represent the real structure of pure germanium cluster with 18 atoms^13,14^.

Here, we show the successful isolation and structural characterization of a germanium cluster Ge_24_^4−^ (1a) featuring an extended prolate structure with fused three-fold faces. Our theoretical calculations show that Ge_24_^4−^ consists of three independent local sigma-aromatic fragments, which is the reason for the collapse of the symmetry and the formation of a prolate structure. This result helps us understand why carbon structures are so different from silicon and germanium ones. Such a model of Ge_24_^4−^ reveals the structural features of medium-sized germanium clusters providing solid prospects for further rationalization of larger species.

## Results

### Preparation of the anionic Ge_24_^4−^ cluster

The title complex [K(2,2,2-crypt)]_4_Ge_24_ (1) was synthesized by mild oxidation of K_12_Ge_17_ using excess Co(dppe)Cl_2_ in ethylenediamine solution at 55 °C. After being layered with toluene for 5 weeks, black block-like crystals occurred on the wall of a reaction test tube in an approximate 25% yield based on K_12_Ge_17_. The structure of 1 was characterized by X-ray diffraction analysis, in which some restraints (SIMU, ISOR and/or DFIX for one K atom and related C, N, O atoms on 2,2,2-crypt) were used in the refined process for better building the model of corresponding [K(2,2,2-crypt)]^+^ fragment. The Co(II) complex of Co(dppe)Cl_2_ was used as a mild oxidizing agent here, which played a crucial role in the formation of a large title cluster. Similarly, the oxidation reactions of Zintl ions could be observed in the previous cluster formation, such as ten-vertex *closo*-E_10_^2−^ (E = Ge/Pb) ^15,16^ and [Ge_10_Mn(CO)_4_]^3−^ ^17^, as well as larger Ge_18_[Si(SiMe_3_)_3_]_6_^14^ and coupling [Ge_9_ = Ge_9_ = Ge_9_]^6−^ ^11^ in which Fe(II) salt and organic reagent like PPh_3_ serve as oxidizing agents, respectively. Furthermore, the redox chemistry involving Co(dppe)Cl_2_ was presented in the synthesis of silyl-protected [Co(dppe)_2_][Ge_9_{Si(SiMe_3_)_3_}_3_] where the Co(II) reagent was reduced by excess K[Ge_9_{Si(SiMe_3_)_3_}_3_] as one counter cation [Co(dppe)_2_]^+^^18^. Such behavior may be useful to understand the role of Co(dppe)Cl_2_ in the synthesis of Ge_24_^4−^ cluster. Besides, several Co-centered cluster species have been also prepared by reactions with different Co complexes, such as [Co@Ge_10_]^3−^ ^19^, [Co_2_@Ge_16_]^4−^ ^20,21^, [Co@M_12_]^3−^ (M = Ge/Pb)^22,23^, [Co@Sn_6_Sb_6_]^3−^ and [Co_2_@Sn_5_Sb_7_]^3−^ ^24^. Interestingly, the [Co_2_@Ge_16_]^4−^ anion contained two types of cluster units (α and β form) and could be obtained by using different Co reagents, Co(PPhEt_2_)_2_(mes)_2_ and [{(ArN)_2_C*t*Bu}Co(η^6^-toluene)]. Unlike the synthesis of [Co(dppe)_2_][Ge_9_{Si(SiMe_3_)_3_}_3_] or [Co@Ge_10_]^3−^, the related reduced products containing Co element failed to be observed or isolated from the en/tol solution. The as-synthesized 1 could not be reproduced by using other cobalt reagents such as CoMes_2_ or tuning down the reaction temperature, otherwise, only some small Ge clusters such as [K(2,2,2-crypt)]_2_Ge_9_ and [K(2,2,2-crypt)]_2_Ge_5_ were afforded.

### Experimental characterization of Ge_24_^4−^ cluster

Energy-dispersive X-ray spectroscopy (EDX, Supplementary Fig. 8) displayed the composition of 1, including only two (semi)metal elements of K and Ge, which is in good agreement with the calculated values. Electrospray‐ionization mass spectrometry by dissolving crystals of 1 in DMF solution indicated besides small fragment of {[K(2,2,2-crypt)][Ge_10_]}^−^, only the corresponding weak signal of parent cluster was observed at *m/z* = 2989.7936 for {[K(2,2,2-crypt)]_3_[Ge_24_]}^−^ due to the inevitable decomposition during the experiments.

**Fig. 1:**
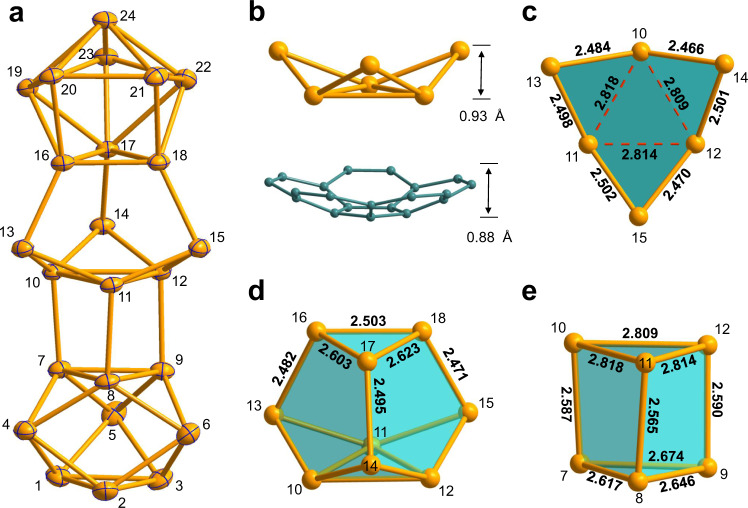
Structures of the Ge_24_^4−^ cluster and its selected fragments. **a** ORTEP representation of cluster Ge_24_^4−^ (1a) at 50% probability. **b** The contrast of bowl-shaped Ge_6_ fragment (top, Ge10-Ge15) with bowl depth of 0.93 Å and corannulene C_20_H_10_ (bottom) with ~0.88 Å. **c** The bowl-shaped Ge_6_ fragment shown from a vertical view. **d** View of Ge_9_ cage (Ge10-Ge18). **e** The distorted prism Ge_6_ fragment consisting of a triangle of Ge7–9 and an extended triangle of Ge10–12. All selected bond lengths are given in Å. The Ge and C atoms are drawn in yellow and blue, respectively.

As shown in Fig. 1a, the overall structure of Ge_24_^4−^ is prolate with an aspect ratio of nearly 3:1 and can be divided into four different polyhedron sections, including a *D*_3h_-symmetric Ge_9_ cage (unit-1, Ge1–9), distorted prism (unit-2, Ge7–12), second peculiar Ge_9_ cage (unit-3, Ge10–18) and the third distorted *C*_4v_-symmetric Ge_9_ cage (unit-4, Ge16–24). This prolate geometry is similar to the ligand-protected tin cluster Sn_20_(Si^t^Bu_3_)_10_Cl_2_ with raspberry-like arrangement of smaller Sn_10_ units, which is formed by the disproportionation reaction of a Sn(I) halide^25^. In light of the structural feature, cluster 1a exhibits a larger prolate structure compared with previous ten-vertex *closo*-E_10_^2−^ (E = Ge/Pb)^15,16^ and [Ge_10_Mn(CO)_4_]^3−^ ^17^ which are formed by similar soft oxidation from basic E_9_ (E = Ge/Pb) units. Such atomic arrangement in 1a is different from the oxidative coupling forms of (Ge_9_)_n_^10–13^. In this sense, Ge_24_^4−^ may undergo a more complex growth pattern. The attempt using the K_4_Ge_9_ as a precursor failed to obtain the title compound under parallel experimental conditions. In contrast to smaller Ge_10_ species, the formation of the title cluster 1a may require a downsizing and further combination of additional Ge_9_ units. The effect of Ge_4_ units from K_12_Ge_17_ is still unclear in the formation of cluster Ge_24_^4−^. Compared with the binary [Au_3_Ge_45_]^9−^ ^26^, the Ge_24_^4−^ cluster represents a medium-sized Ge cluster without doped transition metals. Furthermore, except for the similar structural characteristic from one Ge_9_ of unit 4 and central Ge_6_ fragment, the Ge_24_^4−^ cluster exhibits another type of coordinated Ge_9_ unit to the central Ge_6_ fragment, which is different from the [Au_3_Ge_45_]^9−^ due to the effect of Au atoms.

From another perspective, the Ge_24_^4−^ cluster could also be described as two-terminal Ge_9_-units bridged via a Ge_6_ central fragment along exo-bonds to triangular faces. In this sense, the central bowl-shaped Ge_6_ fragment plays a crucial role in the formation of 1a, and it is suggested as a growth-trigger in the evolution towards larger species. Furthermore, it is also likely to affect the shapes of two terminal Ge_9_-cages by the different coordination fashions. The whole structure can be also viewed as three connected Ge_9_-units involving a terminal cage and fused nine-membered cages sharing three atoms, providing many flavors of the Ge Zintl-ion chemistry in a single molecular structure, able to coincide under similar experimental conditions. Apart from the K_4_Ge_9_, the K_12_Ge_17_ was also used as the source of Ge_9_ unit and related examples have been reported, such as [Ge_9_-Ge_9_]^6−^ (with ZnCp*_2_, Cp*= pentamethylcyclopentadienyl)^27^, (NHC^Dipp^M)_2_{η^3^-Ge_9_(Si(TMS)_3_)_2_} (M = Cu/Ag/Au)^28^, [Ph_2_Bi-(Ge_9_)-BiPh_2_]^2−^ ^29^.

The bowl-shaped Ge_6_ fragment (Fig. 1b) is reminiscent of similar organic molecules corannulene (C_20_H_10_)^30^ or sumanene (C_21_H_12_)^31^, a fullerene fragment, with a curved molecular surface. In contrast, the bowl depth of the Ge_6_ fragment is 0.93 Å, which is close to corannulene (~0.88 Å)^32^. As shown in Fig. 1c, the central triangle (dotted lines) in the Ge_6_ fragment has elongated Ge-Ge distances of av. 2.813 Å like in [Au_3_Ge_45_]^9−^ ^26^, which is remarkably longer than other Ge-Ge bonds with an average length of 2.487 Å. Furthermore, the Ge_6_ bowl combines with a neighboring Ge_3_ face from unit-4 by three Ge-Ge bonds (2.471–2.495 Å) to form an interesting nine-atom cage (Fig. 1d) in which two staggered Ge_3_ faces lead to three almost identical edge-sharing pentagons. In unit-1, the Ge-Ge distances (2.5304(16)-2.6739(16) Å) are in the expected range^33^ and lengths of the prisms (Ge1–Ge7, 2–8, 3–9: 2.8084(16)–2.8744(16) Å) are elongated compared with those (2.71–2.73 Å) in bare *D*_3h_-[Ge_9_]^2–^ cluster^34^. Additionally, the extended bottom face of the central Ge_6_ fragment coordinates to the triangle face of unit-1 through three *exo* Ge-Ge bonds (av. 2.581 Å) forming a distorted triangular prism (Fig. 1e). In contrast to *D*_3h_-unit-1, unit-4 exhibits a largely distorted *C*_4v_-structure with a broader range of Ge-Ge contacts (2.4960(14)-2.8598(15) Å).

### Computational studies

**Fig. 2:**
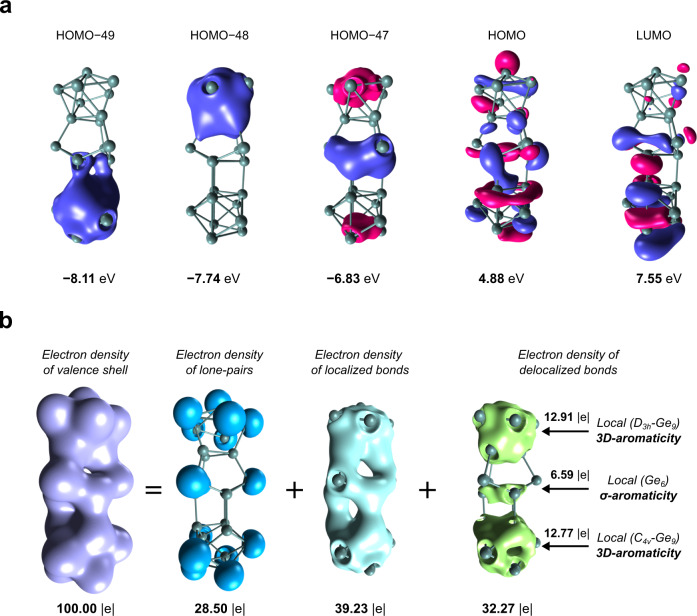
The selected valence molecular orbitals and chemical components of the electron density of Ge_24_^4−^. **a** Selected lowest-lying and frontier valence molecular orbitals in the Ge_24_^4−^ cluster. Different phases of molecular orbitals are represented with different colors. Positive: magenta; negative: purple. **b** The chemical components of the valence-electron density of Ge_24_^4−^ with the corresponding electron populations from the EDDB method. Isosurface value is set at ±0.015 |e|.

To understand the reason for the stability and geometrical features of the Ge_24_^4−^ cluster we performed density functional theory (DFT) calculations^35,36^. The details of theoretical calculations are given in the methods section of this manuscript. The optimized geometry resembles all structural features that were found in the X-Ray experiment. The average Ge-Ge distance of the optimized structure is $\sim$0.07 Å longer than the experimental one, which is a common deviation for the calculation of highly charged Zintl ions with DFT methods. A high HOMO-LUMO gap (2.67 eV) was found for the optimized cluster indicating its remarkably high stability, while shapes of three lowest-lying valence molecular orbitals show certain lack of global aromaticity as the extent of electron delocalization is restricted to three disjoint fragments, D_3h_-Ge_9_ (HOMO-49), C_4v_-Ge_9_ (HOMO-48), and Ge_6_ (HOMO-47) (Fig. 2a). To evaluate whether and to what extent these characteristic delocalization patterns survive the interference with all the remaining valence molecular orbitals, we performed the Electron Density of Delocalized Bonds (EDDB) analysis^37^. The EDDB is a part of the state-of-the-art theoretical method combining different quantum-chemistry and information-theory techniques to decompose the valence-electron density of a molecule into density layers representing chemical entities such as lone pairs, localized (Lewis-type) bonds, and delocalized (‘resonating’) bonds^38^. The results of the EDDB analysis clearly show that 28.5% of the valence-shell electrons do not participate in chemical bonding giving rise to fifteen (4s-type) lone-pairs, about 39.2% of the valence-shell electrons is involved in the Lewis-type Ge-Ge σ-bonding, while the remaining electrons are delocalized in full accordance with topology of HOMO-49, HOMO-48, and HOMO-47, thus marking three independent locally aromatic units: two 3D-aromatic Ge_9_ cages and a single σ-aromatic Ge_6_ fragment (Fig. 2b, Supplementary Movie 1). We note that the presented case is different from cylindrical aromaticity^39^, or organic cages with antiaromatic circuits stacked to each other^40^ since three independent aromatic fragments are aligned together, preserving their individual aromatic properties. The average contribution of each germanium atom in the Ge_24_^4−^ cluster to the electron delocalization is 1.43 |e| , 1.42 | e | , and 1.10 | e| in units D_3h_-Ge_9_, C_4v_-Ge_9_, and Ge_6_, respectively, which is even higher than in the archetypical aromatic system—benzene, where each of the sp^2^-hybridized carbon atoms contributes to the aromatic ring 0.89 | e| and 0.10 | e| through π- and σ-channel, respectively^37^. All this may account for crucial role of the composite aromatic stabilization in the Ge_24_^4−^ cluster, and the lack of effective s-p atomic-orbital hybridization, especially in the Ge_9_ 3D-cages, seems to significantly increase the ability to charge delocalization.

**Fig. 3:**
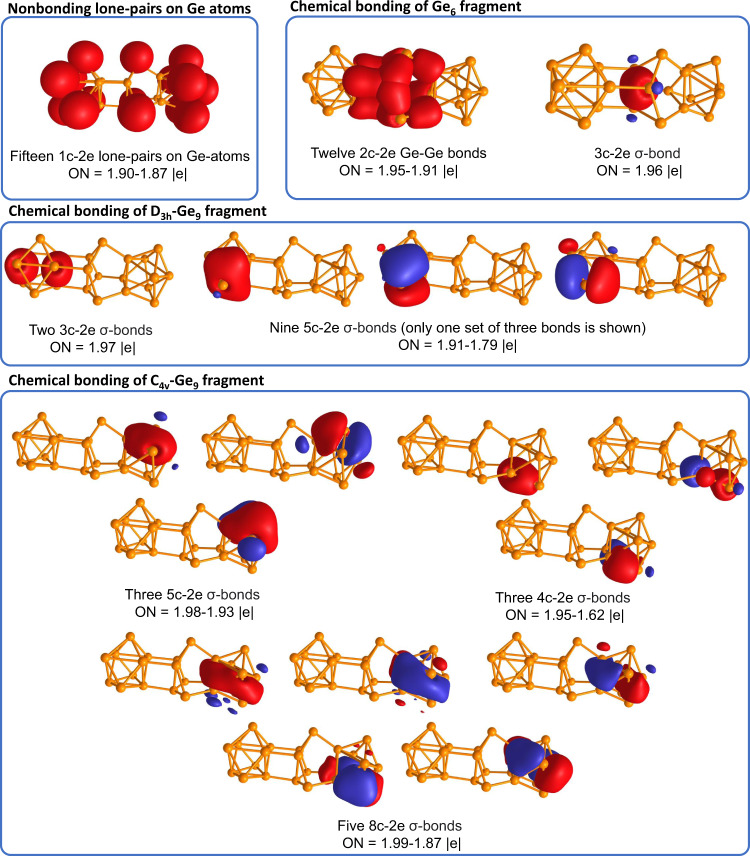
Chemical bonding pattern of the Ge_24_^4−^ cluster. Different phases of bonding elements are represented with different colors. Positive: red; negative: blue.

For a more in-depth and systematic study of the chemical bonding in the synthesized cluster, we performed the Adaptive Natural Density Partitioning (AdNDP) analysis^41,42^. The AdNDP is an electron-localization technique that partitions the natural density of the system and reproduces the most occupied localized bonding elements. The results of the analysis are shown in Fig. 3. Considering one-center two-electron (1c-2e) elements, AdNDP found fifteen s-type lone-pairs with high occupation number values (ON = 1.90–1.87 | e | ) on Ge atoms. Chemical bonding of the middle part of the cluster majorly consists of classical 2c-2e Ge-Ge σ-bonds with ON = 1.95–1.91 | e| and describes a bonding between Ge_6_ and two Ge_9_ fragments. Highly occupied delocalized 3c-2e σ-bond (ON = 1.96 | e | ) governs the bonding within the Ge_6_ fragment and stabilizes the bowl-like Ge_6_ structure. Chemical bonding of the D_3h_-Ge_9_ fragment consists of two 3c-2e σ-bonds with ON = 1.97 | e| and nine 5c-2e σ-bonds (three bonds per each Ge_5_ cap) with ON = 1.91–1.79 | e | . The collection of such delocalized bonding elements possesses spherical-like shielding cones as was shown in our previous studies^43,44^. Analogically, the chemical bonding of the C_4v_-Ge_9_ fragment consists of three delocalized bonding regions resulting in three 5c-2e bonding elements within the Ge_5_ cap (ON = 1.98–1.93 | e | ), three 4c-2e bonding elements within the Ge_4_ square (ON = 1.95–1.62 | e | ), and five 8c-2e bonding elements within the Ge_8_ antiprism (1.99–1.87 | e | ). We note that the low occupation number of 4c-2e bonding element could be increased up to 1.94 | e| with the inclusion of all atoms of Ge_8_ antiprism (Supplementary Fig. 12). A similar situation was earlier described for the isolated C_4v_-Ge_9_^4−^ cluster^45^. That assignment does not change the overall chemical bonding picture. Shapes of the found bonds and numbers of electrons on the fragments that agree with the Hückel’s (4n + 2) electron counting rule render two Ge_9_ fragments locally σ-aromatic^45^. From the chemical bonding analysis described above, we can expect the presence of three independent aromatic regions from the C_4v_-Ge_9_, Ge_6_, and D_3h_-Ge_9_ fragments, in full agreement with theoretical results obtained by the EDDB method.

**Fig. 4:**
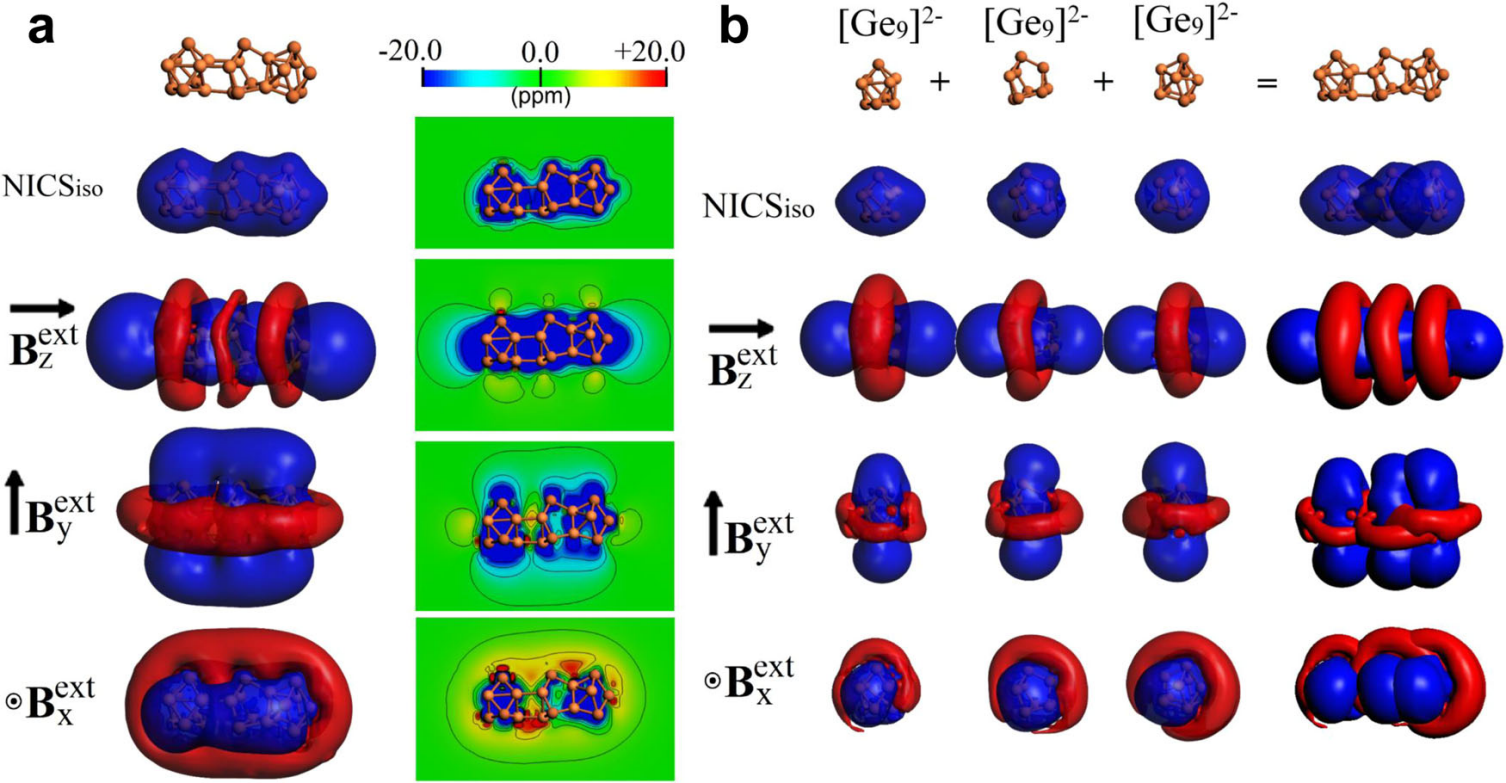
Contour plots and isosurfaces of magnetic response of the Ge_24_^4−^ cluster and various Ge_9_^4−^ units. **a** Isosurface and contour plot representation for NICS_iso_ and certain orientations of the external field for Ge_24_^4−^. Isosurface value is set at ±3.0 ppm. **b** Isosurface representation for NICS_iso_ and certain orientations of the external field for the three isolated Ge_9_^4−^ units, as found in Ge_24_^4−^. Isosurface value is set at ±3.0 ppm. Blue surface: shielding; Red surface: deshielding.

In order to further explore the aromatic characteristics of 1a, the magnetic criteria of aromaticity was employed (Fig. 4a)^46–48^. The isotropic term, given by NICS_iso_ three-dimensional grids, similar to isochemical shielding surface (ICSS) maps, shows a continuous shielding region along with the entire structure. Significantly, under different orientations of the applied field, the shielding cone characteristics were found. In contrast to planar aromatic species for which shielding cones are enabled only when the field is oriented perpendicular to the ring^49^, we found the presence of three cones merged together for any direction of the applied field^48,50^. With the field-oriented along the axis containing all the three cluster fragments (*i.e*. external field oriented along with the *z*-axis, **B**_z_^ind^), a formation of three-overlapped shielding cones centered at each Ge-fragment is observed. For perpendicular orientations (*i.e. y*- and *x*-axis, **B**_y_^ind^ and **B**_x_^ind^ respectively), the three shielding cones are aligned similar to the anthracene molecule, which features three fused aromatic rings as depicted in previous works (Supplementary Fig. 10)^51,52^. Such features are retained under arbitrary orientations of the applied field, denoting how the three adjacent shielding cones evolve under rotation (Supplementary Fig. 11).

Next, we explore the characteristics of each aromatic unit. To represent Ge_6_ bowl-like structure, a Ge_9_ cluster with a shared triangular face of *C*_4v_-Ge_9_ was chosen. Interestingly, despite of fragments’ different shapes, each isolated fragment exhibits similar characteristics to spherical aromatic species with a continuous shielding region from NICS_iso_, and shielding cone characteristics under different orientations of the field (Fig. 4b)^49^. Noteworthy, the overlap between the aromatic characteristics of the three isolated Ge_9_^4−^ clusters largely resembles the behavior of the overall Ge_24_^4−^ cluster supporting that after aggregation involving both exo-bonds and face-fusion schemes, each Ge_9_ unit meets the electronic distribution requirements to behave as spherical aromatics. Hence, Ge_24_^4−^ can be viewed as a linear trimer built-up by related aromatic clusters, exhibiting different shapes and aggregation schemes.

## Discussion

The synthesis and characterization of Ge_24_^4−^ cluster in a solid-state is a missing chain link between small germanium clusters and bulk solid-state germanium. It confirms the prolate structure that was predicted computationally in a gas phase for neutral germanium species^4–6^, providing an explicit structural characteristic of a medium-sized Ge cluster. High symmetry collapse in Ge_24_^4−^ occurs due to the presence of multiple local aromaticity and lack of s-p hybridization in Ge. The formation of three independent aromatic units shed light on the reason for the formation of low-symmetric prolate structure. We expect that this kind of aromatic units’ aggregation will be found in many cluster chemical compounds made in the future. We believe that further investigation of the transition from atomic clusters to bulk materials will bring an understanding and a significant advancement for materials design with a target physical property.

## Methods

### Materials and methods

All manipulations and reactions were performed under a dry nitrogen atmosphere in glove box. Ethylenediamine (Aldrich, 99%) and DMF (Aldrich, 99.8%) used in experiments were freshly distilled by CaH_2_ prior to use. Toluene (Aldrich, 99.8%) was distilled from sodium/benzophenone under nitrogen and stored under nitrogen. 2,2,2-crypt (4,7,13,16,21,24-Hexaoxa-1,10-diazabicyclo (8.8.8) hexacosane, purchased from Sigma-Aldirich, 98%) and Co(dppe)Cl_2_ (purchased from Alfa Aesar, ≥97%) were dried in vacuum for 12 h prior to use. According to reported literature^53^, the precursor K_12_Ge_17_ was synthesized by heating a stoichiometric mixture of the elements (K: 551 mg, Ge: 1.45 g; K: + 99%, Ge: 99.999%, all from Strem) at a rate of 150 °C per hour to 900 °C and keeping it for 3 days in sealed niobium containers closed in evacuated quartz ampules. The furnace was slowly cooled to room temperature at a rate of 100 °C per hour.

#### Synthesis of [K(2,2,2-crypt)]_4_Ge_24_ (1)

K_12_Ge_17_ (170 mg, 0.100 mmol) and 2,2,2-crypt (160 mg, 0.424 mmol) were dissolved in 3 mL en in a reaction vial and stirred for 10 min. Co(dppe)Cl_2_ (63.4 mg, 0.120 mmol) was added and stirred for 6 h at 55 °C. The resulting brown-red solution was filtered with standard glass frit and layered with 4 mL toluene. About 35 days later, black block-like crystals 1 were observed in the test tube (25% yield based on K_12_Ge_17_).

#### X-ray diffraction

Suitable crystal from 1 was selected for X-ray diffraction analysis. Crystallographic data was collected on Rigaku XtalAB Pro MM007 DW diffractometer with graphite monochromated Mo Kα radiation (λ = 0.71073 Å). The structure of crystal 1 was solved using direct methods and then refined using SHELXL-2014 and Olex2^54–56^. All the non-hydrogen atoms were refined anisotropically. All hydrogen atoms of organic groups were rationally placed by geometrical considerations. We used the PLATON SQUEEZE procedure to remove the solvent molecules which could not be modeled properly^57^. We refined the structure by using the rational restraints of anisotropy (SIMU, ISOR, DFIX for K-crypt fragments) and omitted the most disagreeable reflections.

#### Electrospray ionization mass spectrometry (ESI-MS)

Negative ion mode ESI-MS of the DMF solution of crystals of 1 was measured on an LTQ linear ion trap spectrometer by Agilent Technologies ESI-TOF-MS (6230). The spray voltage was 5.48 kV and the capillary temperature was kept at 300 °C. The capillary voltage was 30 V. The samples were prepared inside a glovebox and very rapidly transferred to the spectrometer in an airtight syringe by direct infusion with a Harvard syringe pump at 0.2 mL/min.

#### Energy dispersive X-ray (EDX)

EDX analysis on the title cluster 1 was performed using a scanning electron microscope (FE-SEM, JEOL JSM-7800F, Japan). Data acquisition was performed with an acceleration voltage of 20 kV and an accumulation time of 60 s.

#### Powder X-ray diffraction

Powder X-ray diffraction (PXRD) data were collected on a Rigaku diffractometer using Cu Ka radiation (λ = 1.5418 Å). The sealed samples were scanned for every 0.01° increment over the Bragg angle range of 10 − 80°.

#### Quantum chemical calculations

##### Magnetic response analysis

Geometry optimizations and subsequent calculations were performed using scalar relativistic DFT methods employing the ADF code with the all-electron triple-ζ Slater basis set plus the double-polarization (STO-TZ2P) basis set in conjunction with the PBE0 functional^36,58,59^. In order to evaluate the induced field (**B**^ind^) upon an external magnetic field (**B**^ext^) at the molecular surroundings, according to **B**_*i*_^ind^ = −σ_*ij*_**B**_*j*_^ext^^46,60–63^, the nucleus-independent shielding tensors (σ_ij_)^46,63,64^ were calculated within the GIAO formalism, employing the OPBE^59,65,66^ functional and the all-electron triple-ζ Slater basis set plus the double-polarization (STO-TZ2P), placed in a three-dimensional grid. Relativistic effects were considered through the ZORA Hamiltonian^67^, ensuring an equal footing treatment of different clusters. For convenience, the *I* and *j* suffixes are related to the x-, y- and z-axes of the molecule-fixed Cartesian system (*i, j* = x, y, z). The values of B^ind^ are given in ppm in relation to B^ext^.

##### Chemical bonding analysis

Geometry optimization and frequency calculations were performed using Gaussian 16 software at the PBE0/Def2-QZVP level of theory^36,59,68^. To analyze the extent of electron delocalization in the investigated species, we performed the electron density of delocalized bonds (EDDB) calculations^37,38^; to identify and characterize the chemical bonding, we carried out adaptive natural density partitioning (AdNDP) analysis as implemented in the AdNDP 2.0 code^41,42^. The EDDB and AdNDP analyses were performed at PBE0/Def2-TZVP level of theory; previously, the results by both methods have been shown to be insensitive to the size of the basis set used^38,69^.

## Supplementary information

Supplementary Information

Description of Additional Supplementary Files

Supplementary Movie 1

## Data Availability

The additional data that support the findings of this study are available from the corresponding authors on a request. The X-ray crystallographic coordinates for structure reported in this study have been deposited at the Cambridge Crystallographic Data Centre (CCDC), under deposition number 2072965.
