# Supplementary material for: Symmetry collapse due to the presence of multiple local aromaticity in Ge244−
Source: Nat Commun. 2022 Apr 20;13:2149. doi: 10.1038/s41467-022-29626-5 (PMC9021308; doi:10.1038/s41467-022-29626-5)
Supplement: Supplementary file 1 — Supplementary Information [file 41467_2022_29626_MOESM1_ESM.pdf]

Supplementary Materials for

Symmetry Collapse due to the Presence of Multiple Local Aromaticity in Ge_{24}^{4-}

Hong-Lei Xu, Nikolay V. Tkachenko, Dariusz W. Szczepanik, Ivan A. Popov, Alvaro Muñoz-Castro, Alexander I. Boldyrev,* Zhong-Ming Sun*

Content

Section 1: Crystallographic Supplementation. 2
Supplementary Figure 1. Crystals of title compound $[K(2,2,2\text{-crypt})]_4Ge_{24}$ (**1**). 2
Supplementary Table 1. Crystal data and structure refinement for **1**. 2
Supplementary Figure 2. Asymmetric unit of **1**. 3
Supplementary Figure 3. Unit cell of **1**. 4
Supplementary Figure 4. The contrast of sphere-like D_{3h}-Ge_9 moiety and non-classical Ge_9 moiety in anion 1a with different coordination modes. 4
Section 2: ESI-MS Studies 5
Supplementary Figure 5. Overview ESI mass spectrum in negative ion mode recorded immediately upon injection of a fresh solution of crystals **1** in DMF. 5
Supplementary Figure 6. Measured and simulated spectrum of the fragment $\{[K(2,2,2\text{-crypt})][Ge_{10}]\}^-$. 6
Supplementary Figure7. Measured and simulated spectrum of the fragment $\{[K(2,2,2\text{-crypt})]_3[Ge_{24}]\}^-$. 7
Section 3: Energy Dispersive X-ray (EDX) Spectroscopic Analysis 7
Supplementary Figure 8. EDX analysis of **1** (K, Ge). 7
Supplementary Figure 9. Powder X-ray diffraction (PXRD) pattern of "$K_{12}Ge_{17}$". 7
Section 4: Supplementary Computational Data 8
Supplementary Figure 10. Isosurface representation for the induced magnetic field for anthracene molecule 8
Supplementary Figure 11. Isosurface representation for the induced magnetic field for $[Ge_{24}]^{4-}$ under different orientations of the external field. 9
Supplementary Figure 12. AdNDP analysis of C_{4v}-Ge_9 fragment 9
Supplementary Table 2. Cartesian coordinates of optimized cluster. 10

Section 1: Crystallographic Supplementation.

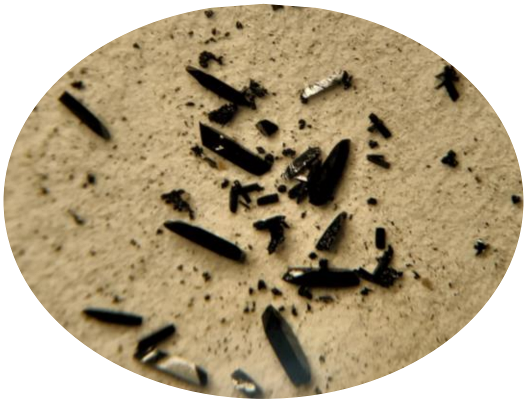

Supplementary Figure 1. Crystals of title compound $[K(2,2,2\text{-crypt})]_4Ge_{24}$ (**1**).

Supplementary Table 1. Crystal data and structure refinement for **1**.

Identification code	**1**
Empirical formula	$C_{72}H_{144}Ge_{24}K_4N_8O_{24}$
Formula weight	3404.50
Temperature/K	100
Crystal system	monoclinic
Space group	$P2_1/n$
a/Å	16.3953(5)
b/Å	16.2793(5)
c/Å	47.2990(14)
α/°	90
β/°	99.429(3)
γ/°	90
Volume/Å^3	12453.7(7)
Z	4
ρ_{calc}g/cm^3	1.816
μ/mm^{-1}	5.883
F(000)	6672.0
Crystal size/mm^3	0.2 × 0.14 × 0.12
2*Θ* range for data collection/°	7.248 to 50
Index ranges	$-19 \leq h \leq 19$, $-19 \leq k \leq 19$, $-56 \leq l \leq 56$
Reflections collected	110915
Independent reflections	21894 [R_{int} = 0.0880, R_{sigma} = 0.0578]
Data/restraints/parameters	21894/877/1189
Goodness-of-fit on F^2	1.097

Final R indexes [I>=2σ (I)]	R_1 = 0.0755, wR_2 = 0.1473
Final R indexes [all data]	R_1 = 0.1012, wR_2 = 0.1560
Largest diff. peak/hole / e Å^{-3}	2.75/-1.05
CCDC	2072965

Many attempts were made to pick more crystals of title compound for x-ray analysis, but failed to obtain better data quality than current one due to the limitation of crystal quality in en solvent. In addition, we also optimized other crystallization conditions by changing the solvents, such as dmf or pyridine, still failed.

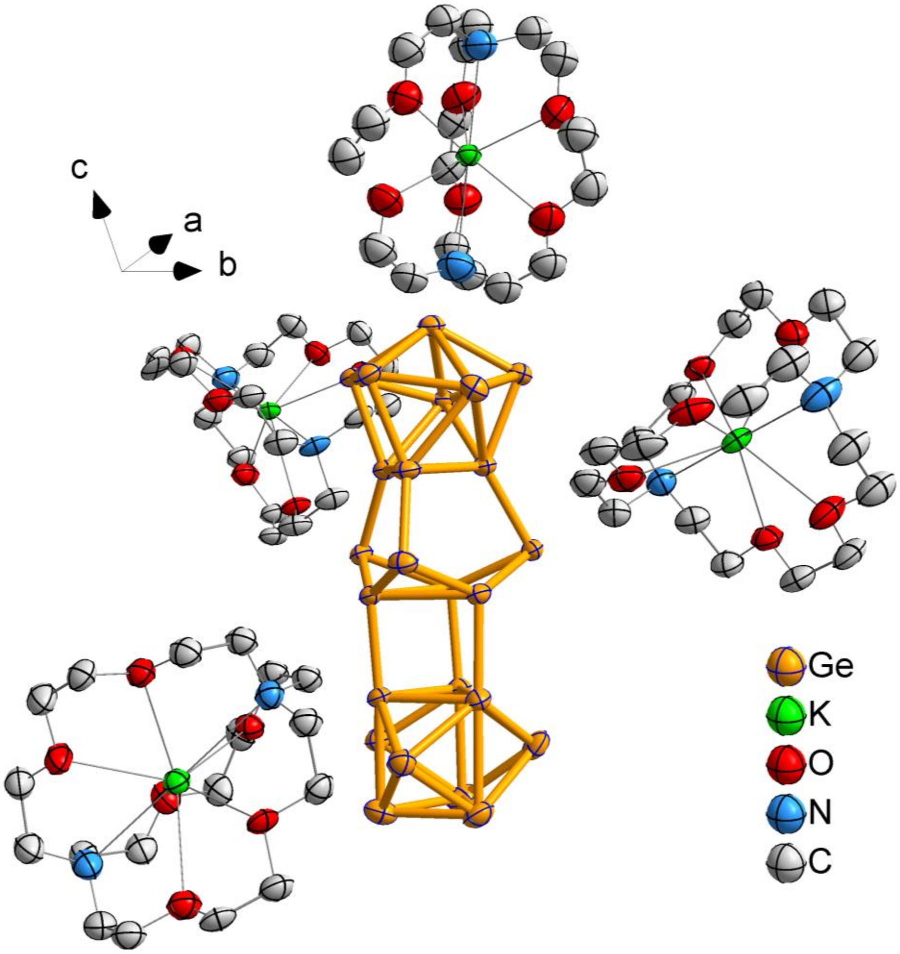

Supplementary Figure 2. Asymmetric unit of **1**. Thermal ellipsoids are drawn at 50% probability. The minor components are omitted for clarity.

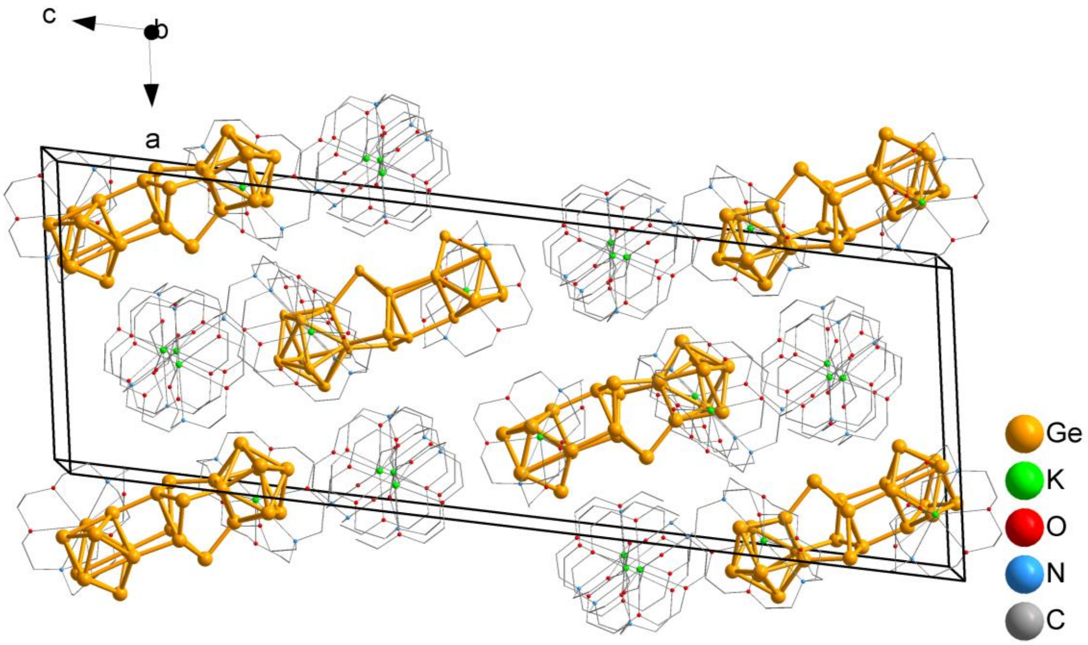

Supplementary Figure 3. Unit cell of **1**. Minor component in the cluster site is omitted for clarity.

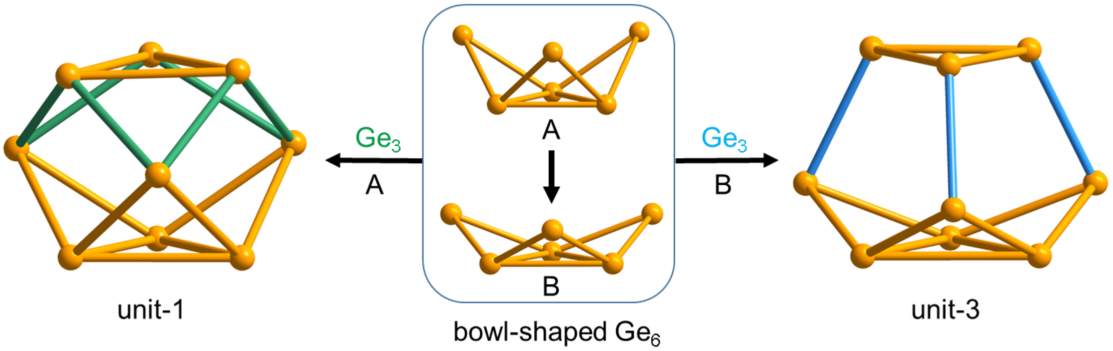

Supplementary Figure 4. The contrast of sphere-like D_{3h}-Ge_9 moiety (left, unit-1) and non-classical Ge_9 moiety (right, unit-3) in anion 1a with different coordination modes.

Section 2: ESI-MS Studies

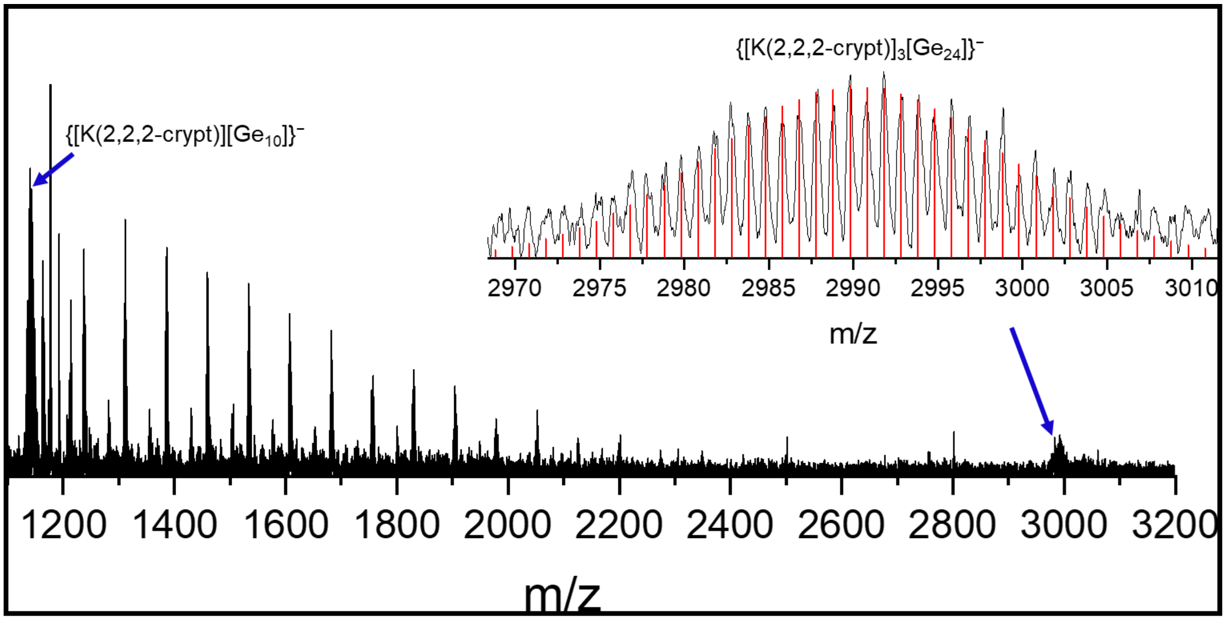

Supplementary Figure 5. Overview ESI mass spectrum in negative ion mode recorded immediately upon injection of a fresh solution of crystals 1 in DMF. Electrospray-ionization mass spectrometry (ESI-MS) on single crystals of **1** was performed to observe the corresponding signal of parent cluster at *m/z*= 2989.7936 for $\{[K(2,2,2\text{-crypt})]_3[Ge_{24}]\}^-$ as well as the signal of small fragment $\{[K(2,2,2\text{-crypt})][Ge_{10}]\}^-$ at *m/z*= 1142.4437. The weak signals especially for the parent cluster fragment were generated by inevitable decomposition during the experiments.

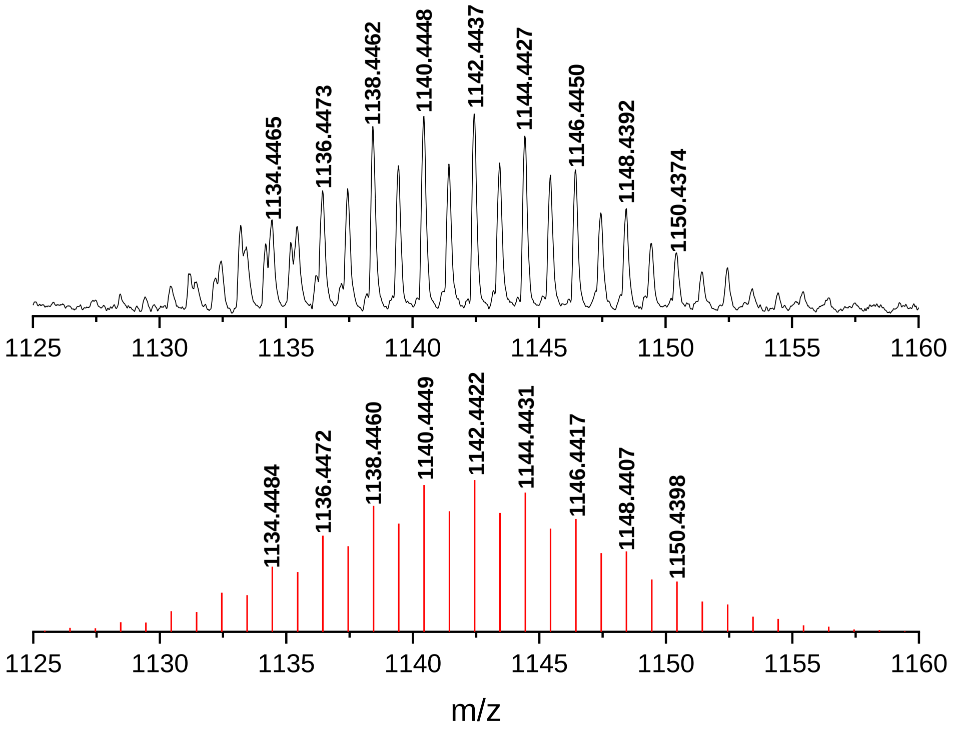

Supplementary Figure 6. Measured (top) and simulated (bottom) spectrum of the fragment $\{[K(2,2,2\text{-crypt})][Ge_{10}]\}^{-}$.

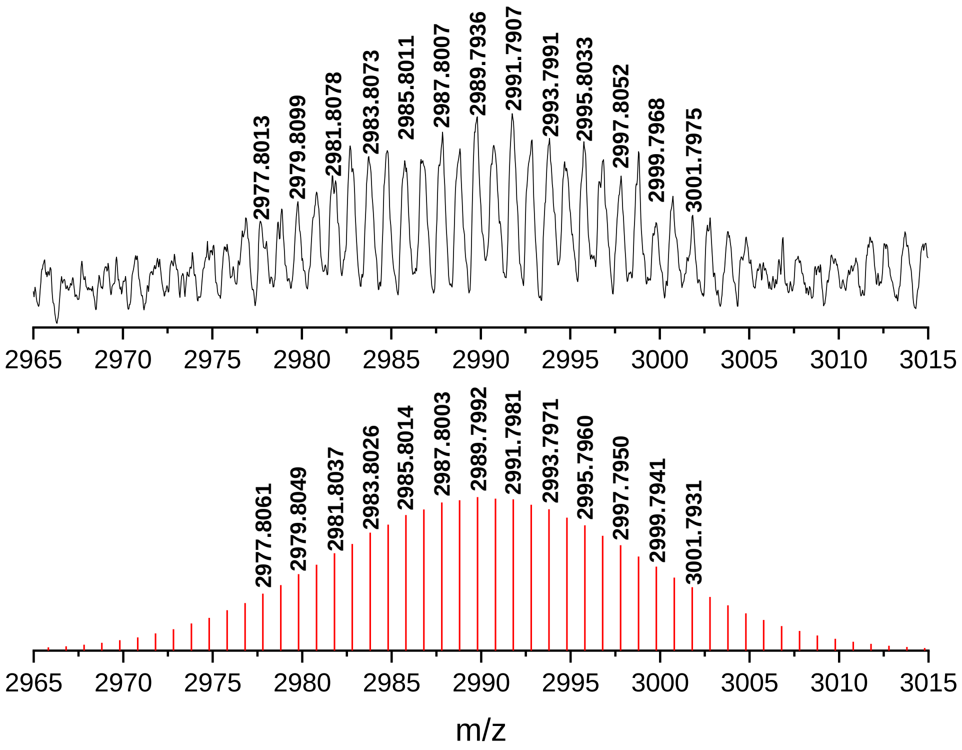

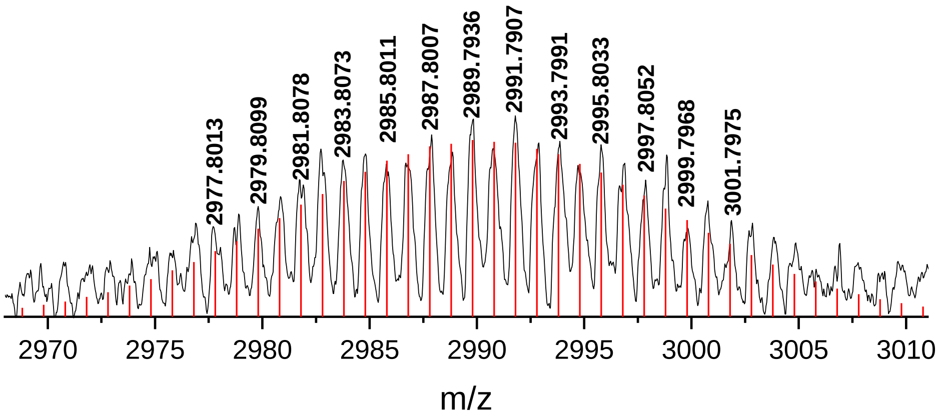

Supplementary Figure 7. Measured (top), simulated (middle) spectrum and their comparison (bottom) of the fragment $\{[K(2,2,2\text{-crypt})]_3[Ge_{24}]\}^-$.

Section 3: Energy Dispersive X-ray (EDX) Spectroscopic Analysis

The EDX revealed the composition of **1**, including only K and Ge but not Co. The result showed the atom% values of K: Ge (~ 8.7 %:91.3 %), which are in good agreement with the theoretical values of K_4Ge_{24} (8.2 %:91.8 %).

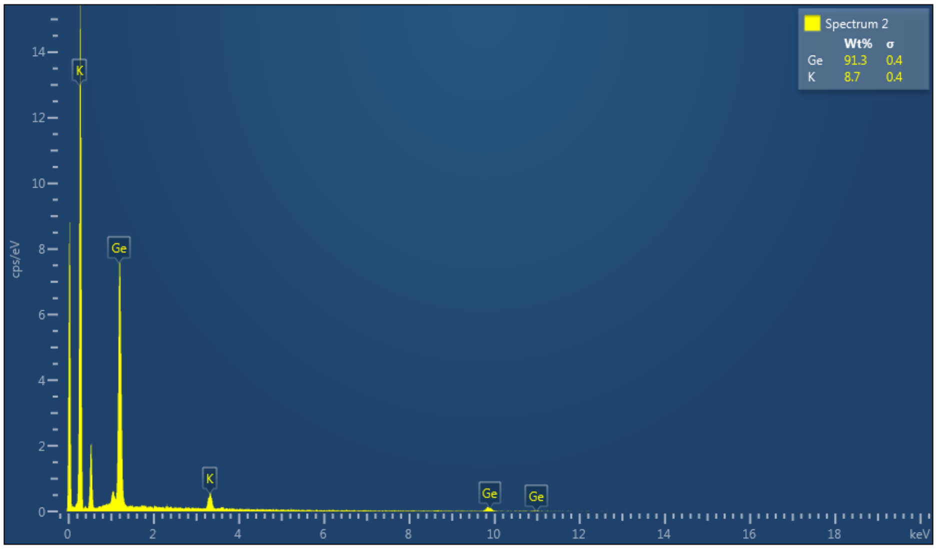

Supplementary Figure 8. EDX analysis of 1 (K, Ge).

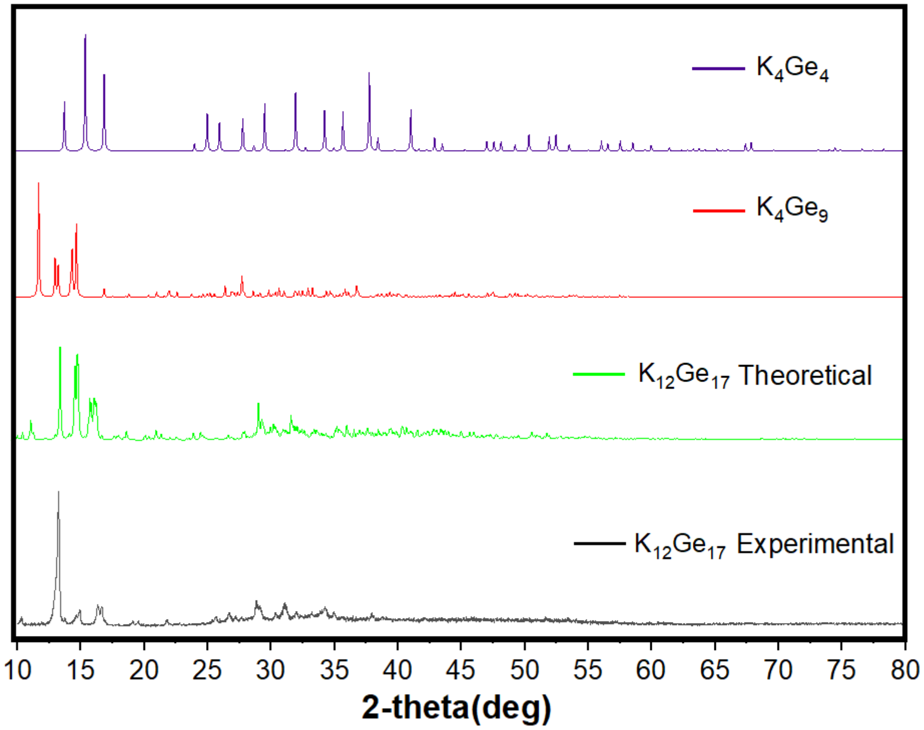

Supplementary Figure 9. Measured powder X-ray diffraction (PXRD) pattern of $K_{12}Ge_{17}$ (black). The simulated patterns of $K_{12}Ge_{17}$ (green), K_4Ge_9 (red), and K_4Ge_4 (purple) were present for reference.

Section 4: Supplementary Computational Data

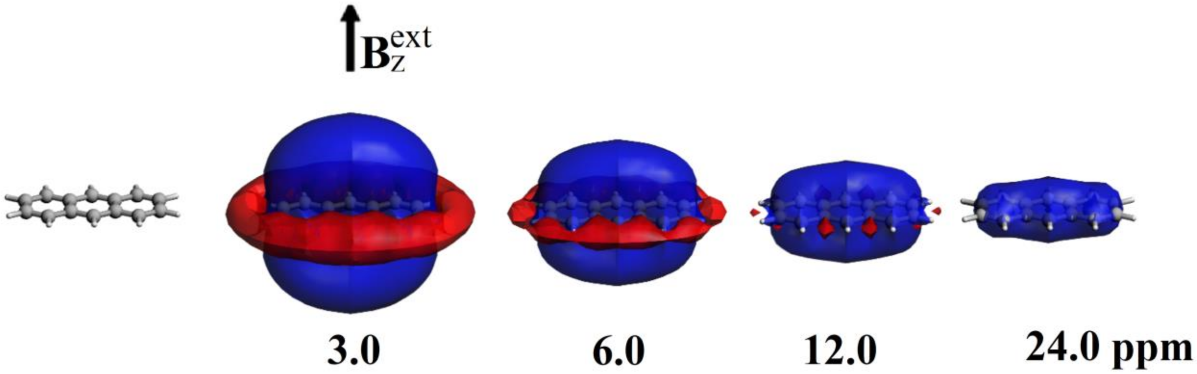

Supplementary Figure 10. Isosurface representation for the induced magnetic field for anthracene molecule under aligned with the z-axis, showing the three shielding response obtained by the three-consecutive rings. Isosurface value set at ±3.0, ±6.0, ±12.0, and, ±24.0 ppm, as noted in the figure.

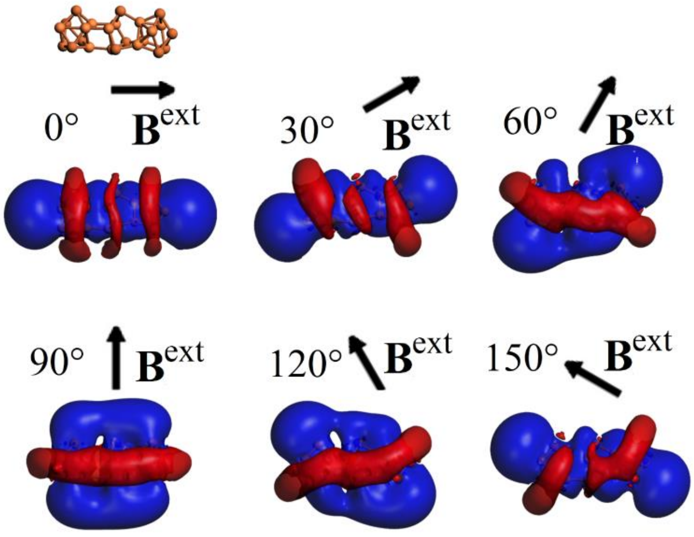

Supplementary Figure 11. Isosurface representation for the induced magnetic field for $[Ge_{24}]^{4-}$ under different orientations of the external field, noted by arrows. Isosurface value set at ±3.0 ppm.

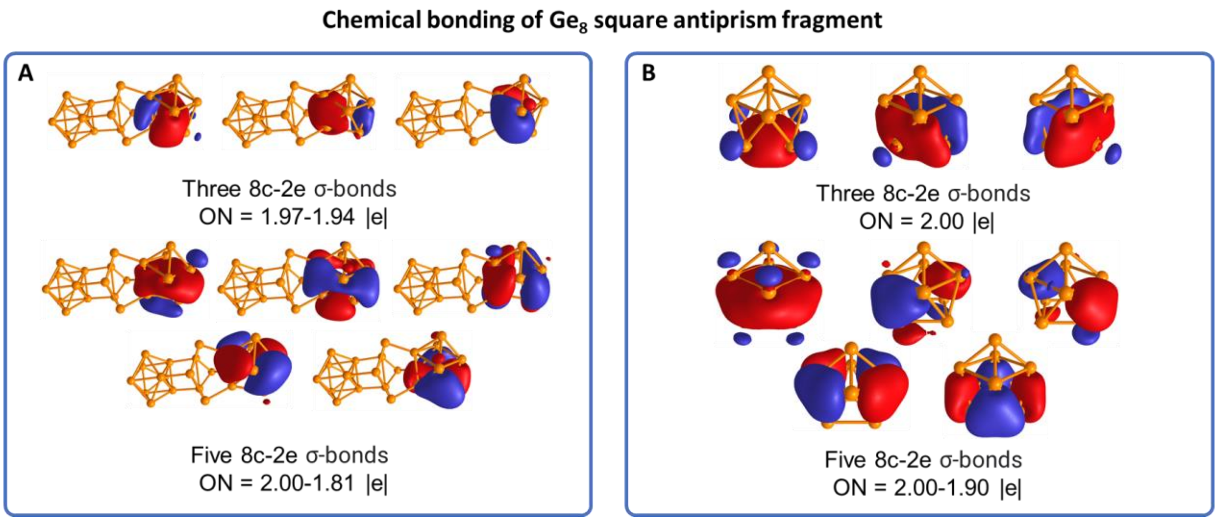

Supplementary Figure 12. AdNDP analysis of Ge_8 antiprism fragment of Ge_{24}^{4-} (A) and C_{4v}-Ge_9^{4-} (B) clusters.

Supplementary Table 2. Cartesian coordinates of optimized cluster.

Ge_{24}^{4-}	PBE0/def2qzvp, NIMAG = 0			
	32	0.418540000	-1.658830000	0.003816000
	32	0.389177000	0.761533000	-1.395618000
	32	-2.800853000	1.355681000	-0.002650000
	32	0.390325000	0.766371000	1.392407000
	32	3.029709000	-1.514251000	0.004727000
	32	-0.607573000	2.594629000	-0.004468000
	32	3.009435000	0.781176000	-1.330995000
	32	-2.766069000	-0.945504000	-1.255222000
	32	-0.530213000	-1.372493000	-2.323637000
	32	-5.184301000	-1.744656000	-1.364101000
	32	-4.615257000	0.757758000	-2.070989000
	32	-2.766708000	-0.940312000	1.259417000
	32	3.011290000	0.787276000	1.331526000
	32	-0.530946000	-1.362849000	2.329659000
	32	-5.087569000	2.390776000	-0.005779000
	32	-4.615633000	0.767375000	2.067113000
	32	4.385157000	-1.219142000	2.164354000
	32	-6.385503000	0.149829000	-0.000715000
	32	4.381035000	-1.228876000	-2.159125000
	32	-5.185058000	-1.738388000	1.371787000
	32	5.897722000	0.797872000	1.318524000
	32	4.356573000	2.519762000	-0.004755000
	32	5.913011000	-1.497609000	0.001325000
	32	5.893707000	0.792873000	-1.326600000
