# Supplementary material for: Symmetry collapse due to the presence of multiple local aromaticity in Ge244−
Source: Nat Commun. 2022 Apr 20;13:2149. doi: 10.1038/s41467-022-29626-5 (PMC9021308; doi:10.1038/s41467-022-29626-5)
Supplement: Supplementary file 2 — Description of Additional Supplementary Files [file 41467_2022_29626_MOESM2_ESM.pdf]

Description of Additional Supplementary Files

Supplementary Movie 1. The chemical components of the valence-electron density of Ge_{24}^{4-}. Electron density of valence shell (EDVS), electron density of lone-pairs (EDLP), electron density of localized bonds (EDLB), and electron density of delocalized bonds (EDDB) are shown. Isosurface values are changing during the movie within the range of 0.010—0.015 |e|.
